# Risk prediction model for postoperative pneumonia in esophageal cancer patients: A systematic review

**DOI:** 10.3389/fonc.2024.1419633

**Published:** 2024-08-05

**Authors:** Yaxin Jiang, Zimeng Li, Weiting Jiang, Tingyu Wei, Bizhen Chen

**Affiliations:** ^1^ Department of Healthcare-Associated Infection Management, The Second Affiliated Hospital of Fujian University of Traditional Chinese Medicine, Fuzhou, China; ^2^ School of Nursing, Fujian University of Traditional Chinese Medicine, Fuzhou, China

**Keywords:** postoperative pneumonia, risk prediction, modelling, esophageal cancer, system review

## Abstract

**Background:**

Numerous studies have developed or validated prediction models to estimate the likelihood of postoperative pneumonia (POP) in esophageal cancer (EC) patients. The quality of these models and the evaluation of their applicability to clinical practice and future research remains unknown. This study systematically evaluated the risk of bias and applicability of risk prediction models for developing POP in patients undergoing esophageal cancer surgery.

**Methods:**

PubMed, Embase, Web of Science, Cochrane Library, Cumulative Index to Nursing and Allied Health Literature (CINAHL), China National Knowledge Infrastructure (CNKI), China Science and Technology Journal Database (VIP), WanFang Database and Chinese Biomedical Literature Database were searched from inception to March 12, 2024. Two investigators independently screened the literature and extracted data. The Prediction Model Risk of Bias Assessment Tool (PROBAST) checklist was employed to evaluate both the risk of bias and applicability.

**Result:**

A total of 14 studies involving 23 models were included. These studies were mainly published between 2014 and 2023. The applicability of all studies was good. However, all studies exhibited a high risk of bias, primarily attributed to inappropriate data sources, insufficient sample size, irrational treatment of variables and missing data, and lack of model validation. The incidence of POP in patients undergoing esophageal cancer surgery ranged from 14.60% to 39.26%. The most frequently used predictors were smoking, age, chronic obstructive pulmonary disease(COPD), diabetes mellitus, and methods of thoracotomy. Inter-model discrimination ranged from 0.627 to 0.850, sensitivity ranged between 60.7% and 84.0%, and specificity ranged from 59.1% to 83.9%.

**Conclusion:**

In all included studies, good discrimination was reported for risk prediction models for POP in patients undergoing esophageal cancer surgery, indicating stable model performance. However, according to the PROBAST checklist, all studies had a high risk of bias. Future studies should use the predictive model assessment tool to improve study design and develop new models with larger samples and multicenter external validation.

**Systematic review registration:**

https://www.crd.york.ac.uk/prospero, identifier CRD42024527085.

## 1 Introduction

Esophageal cancer (EC) is a common malignant tumor of the gastrointestinal tract that originates from the mucosal epithelium of the esophagus. According to data from the National Cancer Center of China, an estimated 224,000 new cases of EC appeared in 2022 (1). Esophagectomy is currently the primary therapeutic option for EC. Despite significant advancements in surgical technology and perioperative care, esophagectomy remains a high-risk procedure, with a postoperative complication rate of 36.2% (2). Among the complications, postoperative pneumonia (POP) is the most common, with an incidence ranging from 8.7% to 28.3% (3). The occurrence of POP prolongs hospitalization and affects patient prognosis and even death. Patients with POP have a higher mortality rate within 1 year than those who do not develop infection after surgery (16.8% *vs.* 21.6%) (4). Therefore, precise and early assessment of individuals at risk of developing pneumonia after EC, coupled with targeted preventive measures against risk factors, is of utmost importance.

Clinical prediction models combine multiple relevant risk factors to estimate the probability of outcome occurrence so that clinical providers can quickly identify and monitor high-risk patients to avoid adverse events. Recently, numerous predictive models have been developed to predict POP occurrence in patients with EC. These models can be used to identify individuals at high risk of POP; however, there may be contradictions between the results of different studies (5, 6). This poses a major dilemma for surgeons in selecting an appropriate treatment. Therefore, a comprehensive review and overview of existing POP models is necessary to clarify their predictive performance, advantages, and disadvantages, among others, to evaluate the best model with the potential for widespread implementation.

Recent systematic reviews have focused on predictive models for pulmonary complications following esophagectomy (7, 8). However, this is not entirely applicable to identifying people at risk of POP. Our study specifically focused on the population at a high risk of POP following surgery among patients with EC. In this study, we analyzed the existing prediction models for the risk of POP after esophagectomy concerning the reports related to the systematic evaluation of prediction models (9) to provide a reliable assessment tool for preventing and controlling POP.

## 2 Methods

### 2.1 search strategy

In order to conduct a comprehensive search, we targeted both Chinese and English databases. Computerized searches the databases included PubMed, Embase, Web of Science, The Cochrane Library, Cumulative Index to Nursing and Allied Health Literature (CINAHL), China Knowledge Network(CNKI), Chinese Science and Technology Journal Database (VIP), Wanfang database, and China Biomedical Literature Database, which were searched from the inception of the databases until March 12, 2024. A combination of subject and free word search was adopted. Search keywords included Esophageal Neoplasms, Esophagectomy, cancer of esophagus, Pneumonia, postoperative pneumonia, lung infection, Risk Assessment, Risk Factors, prediction model, predict*. We also identified additional relevant studies by reviewing the reference lists of the retrieved studies and review articles.

In this systematic review, we employed the PICOTS system recommended by the Critical Appraisal and Data Extraction for Systematic Reviews of Prediction Modelling Studies (CHARMS) (10) checklist. This approach aids in formulating the review’s objectives, search strategy, and inclusion/exclusion criteria (5). The essential components of our systematic review are delineated below:

P (Population): patients operated on for EC.

I (Intervention model): development and/or validation of predictive models for the risk of POP in patients operated on for EC.

C (Comparator): not applicable.

O (Outcome): the primary outcome indicator is the occurrence of POP.

T (Timing): Esophageal cancer after surgery. The prediction was made according to the laboratory indicators and clinical symptoms in the diagnostic criteria of POP.

S (Setting): the intended use of the predictive models is the prediction of the occurrence of POP in patients operated on for EC, to allow for early identification of at-risk populations and targeted preventive measures.

### 2.2 Inclusion and exclusion criteria

Inclusion criteria:

(1) Patients who have been diagnosed with EC and treated with surgery.
(2) The study is about the construction and/or validation of a prediction model for the risk of pneumonia after surgery for esophageal cancer.
(3) An observational study design.
(4) The predicted outcome is POP.
(5) Studies published in Chinese and English.

Exclusion criteria:

(1) Models consisting of only one predictor (e.g., lung function index, sarcopenia, laboratory tests, etc.).
(2) Studies on risk factors analysis were performed only, without the construction of a complete risk model.
(3) The types of studies were reviews, conference papers, etc.
(4) The full text could not be retrieved despite contacting the authors via email.
(5) Duplicated published studies.

### 2.3 Study screening

The retrieved literature was imported into the literature management software. Firstly, duplicate studies were manually identified and removed. Second, titles and abstracts of articles were screened. Finally, their full texts were reviewed after applying the inclusion and exclusion criteria. The reference lists of all eligible studies were also checked to ensure the comprehensiveness of the search. Two researchers independently conducted and cross-checked the study’s screening. In case of disagreement, a third researcher was involved in the discussion to solve the problem.

### 2.4 Data collection

Following the screening process, Data extraction of the identified studies was performed using the CHARMS (10). The information extracted from the selected studies was categorized into two groups: (1) Basic information: first author, year, country, study type, and sample size. (2) Model information: number of models, modeling method, model efficacy, final predictors, selection of variables, model presentation, verification method. Two researchers independently performed and cross-checked data extraction. Any disagreement was resolved with the help of another researcher.

### 2.5 Assessment of risk of bias and applicability

The risk of bias and applicability of the included studies was evaluated using the Predictive Modeling Risk of Bias Assessment Tool (PROBAST) (11). The risk of bias evaluation included 20 questions in 4 domains: study object, predictor, outcome, and data analysis. The applicability evaluation included three domains: study object, predictor, and outcome. When the results of all domains were “low risk of bias,” the result of the risk of bias assessment was “low risk of bias”; when “high risk of bias” was found in ≥1 domain, the result of the risk of bias assessment was “high risk of bias”; when the result of one domain is “unclear,” and the results of the remaining domains are “low risk of bias,” the risk of bias assessment result is “unclear.” The applicability assessment was similar to the risk of bias assessment. The two researchers carried out the literature assessment, and after the independent assessment, they checked each other’s results. Disagreements, if any, in the above process were resolved through discussion. A third researcher could also be consulted if necessary.

### 2.6 Descriptive analyses

Results were summarized using descriptive statistics, which was conducted on the included studies, the establishment of prediction models, and the performance of models.

### 2.7 Patient and public involvement

There was no patient or public involvement in this study.

## 3 Results

### 3.1 Process of article screening

**Figure 1:**
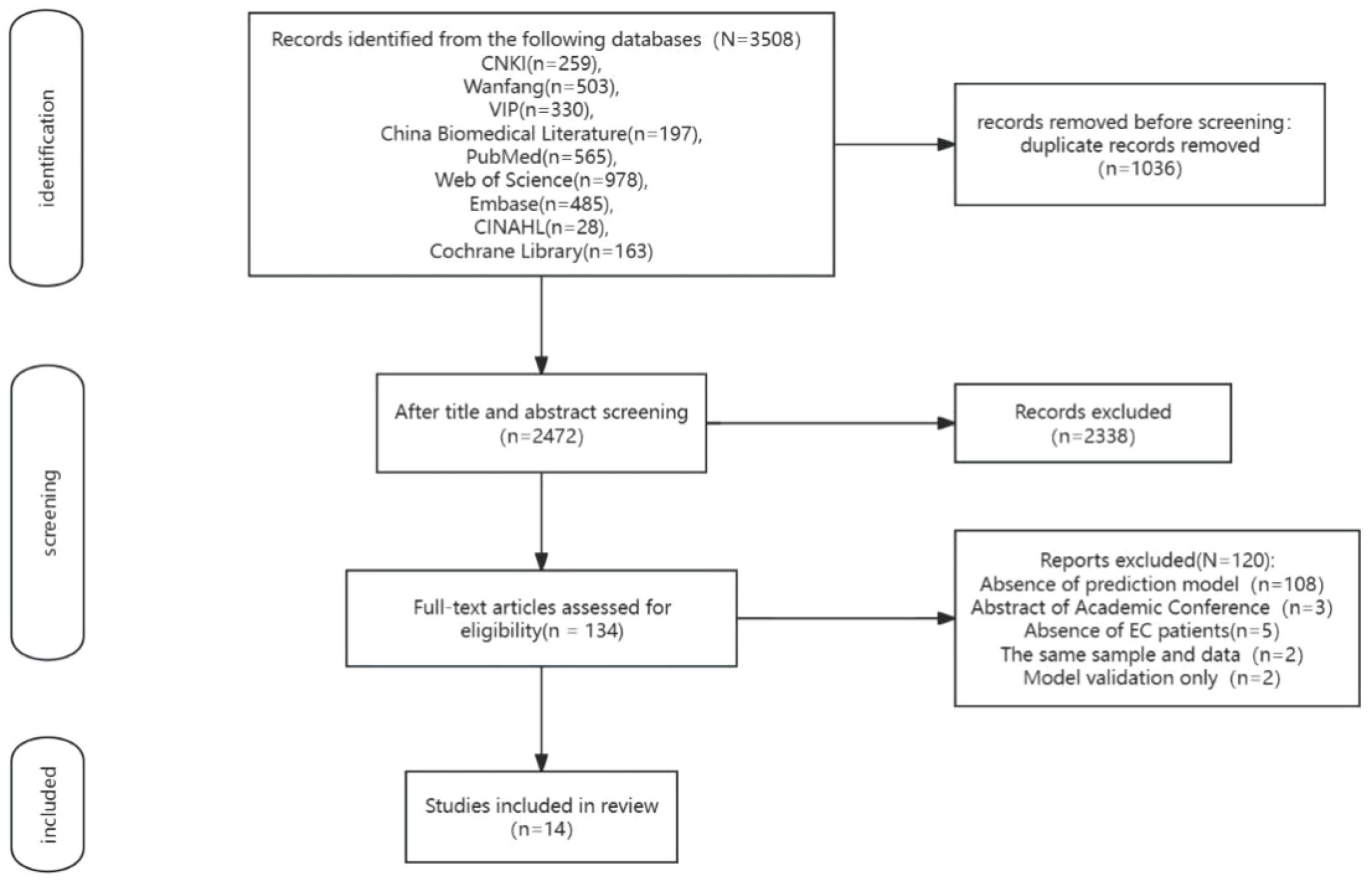
Process of study selection.

The search strategy identified 3508 records, and 1,036 duplicate records were deleted. After title and abstract screening, 2,338 records were excluded. After reviewing the full texts, 120 records were further excluded(108 articles only analyzed risk factors for the development of POP in patients undergoing surgery for EC, three articles were conference papers, five articles had study participants who did not meet the inclusion criteria for this study, 2 duplicated published studies, and two studies only validated the model). Ultimately, 14 articles of literature (12–25) were included in this systematic review. The study selection process is shown in **Figure 1**.

### 3.2 Study characteristics

The year of publication of the 14 included studies was primarily between 2014 and 2023. Twelve were conducted in China (14–25) (nine studies published in Chinese), one from the Netherlands (12) and one from Japan (13). The majority (n=11, 78.57%) of the included studies were retrospective. One Japanese study (13) involved multiple centers, while the others were conducted at single centers. Regarding the study population, two studies (14, 15) focused on EC patients aged 60 years or older. The specific characteristics of the included studies are summarized in **Table 1**.

**Table 1:** General characteristics of the included studies (n =14).

Author(year)	Region	Study design	Participants	POP Definition	POP cases/sample size(%)
Shi Peijun (2023) (16)	China	P	Patients undergoing radical surgery for esophageal cancer	Clinical Diagnostic Criteria for Lung Infections Released by the Chinese Medical Association Respiratory Disease Section (26)	122/548 (22.26%)
Li Cuihua (2023) (23)	China	R	Patients aged>40 years undergoing radical esophageal cancer surgery	Diagnostic Criteria for Hospital-acquired Infections (27)	–
Niu Lingjuan (2023) (24)	China	C	Esophageal cancer patients aged 18–80 years undergoing surgical treatment	Diagnostic Criteria for Hospital-acquired Infections (27)	40/156 (25.64%)
Fan Yixin (2023) (14)	China	R	Patients aged≥60 years undergoing radical esophageal cancer surgery	Clinical Diagnostic Criteria for Lung Infections Released by the Chinese Medical Association Respiratory Disease Section (26)	61/179 (34.08%)
Zhang Yefan (2023) (17)	China	R	Esophageal cancer involving the lower esophagus	Revised Uniform Pneumonia Score(rUPS) (12)	151/396 (38.1%)
Dang Xinchen (2021) (18)	China	R	Patients with stage I-III esophageal cancer without radiotherapy and undergoing radical esophageal cancer surgery	Clinical Diagnostic Criteria for Lung Infections Released by the Chinese Medical Association Respiratory Disease Section (26)	188/644 (29.19%)
Xu Lei (2018) (19)	China	R	18–85years old, stage 0-III primary esophageal cancer who underwent radical esophageal cancer surgery	Diagnostic criteria for pneumonia published by the American Thoracic Society and the Infectious Diseases Society of America (28)	158/806 (19.6%)
Chen Qiongyi (2023) (15)	China	R	Age≥60 years, primary esophageal cancer 0-III, and radical esophageal cancer surgery	Diagnostic criteria for pneumonia published by the American Thoracic Society and the Infectious Diseases Society of America (28)	148/377 (39.26%)
Bi Cen (2022) (20)	China	R	First-time recipients of radical esophageal cancer surgery	Clinical Diagnostic Criteria for Lung Infections Released by the Chinese Medical Association Respiratory Disease Section (26)	54/221 (24.43%)
Wang Wei (2022) (21)	China	R	Patients with esophageal squamous cell carcinoma receiving neoadjuvant immunochemotherapy followed by surgery	Clavien-Dindo Classification (CDC) classification grading system (29)	26/78 (33.33%)
Jin Donghui (2022) (22)	China	R	Persons undergoing esophagectomy	Clavien-Dindo Classification (CDC) classification grading system (29)	111/609 (18.2%)
Van (2014) (12)	Netherlands	P	Patients with esophageal cancer undergoing esophagectomy	Modified Clavien Dindo Classification (MCDC) grade II (29)	70/185 (37.8%)
Ohkura (2020) (13)	Japan	R	Esophagectomy patients	–	1588/10862(14.6%)
Li Shuang (2021) (25)	China	R	Age≥18 years for esophagectomy	Centers for Disease Control and Prevention and National Healthcare Safety Network surveillance definition (30)	131/637 (20.57%)

P, prospective cohort study; R, retrospective cohort study; C, case-control study; -, Unreported.

### 3.3 Basic features of prediction model

Most included studies developed only one model, while four studies (12, 13, 15, 16) developed more than one model, resulting in 30. Notably, the study by Ohkura contributed eight models (13). However, our review focuses only on the most relevant models (POP). Ultimately, our systematic review evaluates 23 models. The sample size across all studies ranged from 78 to 10,862 patients. Only six studies (12, 15, 17–20) reported the number of missing sample cases and addressed them through direct exclusion. In terms of model development, the methodologies used by most studies were logistic regression. Additionally, five studies (16–18, 21, 22) utilized R software for constructing their models, and only 1 study developed a predictive model using machine learning techniques (15). For predictors, the final number of predictors obtained ranged from 2 to 17, with the five most frequently occurring predictors being smoking, age, chronic obstructive pulmonary disease(COPD), diabetes, and open chest surgery method. The basic features information in the prediction model is summarized in **Table 2**.

**Table 2:** Establishment of a POP risk prediction model in patients with esophageal cancer(n =14).

Author (year)	Quantity	Sample size				Modeling methodology	Selection of variables	Predictor	Model presentation
		Total sample size	Build	Verify	Missing data				
Shi Peijun (2023) (16)	2	548	247	247	–	R software	lasso	Age, smoking history, diabetes, underlying lung disease, PNI, PCT, IL-1as sTREM-1, CD4+/CD8+	Nomograph
Li Cuihua (2023) (23)	1	126	74	52	–	Logistic	Multifactor analysis	Smoking, diabetes, chronic respiratory disease, CRP on the first postoperative day, PCT on the first postoperative day	Formulas,nomograph
Niu Lingjuan (2023) (24)	1	156	156	–	–	Logstic	Multifactor analysis	Age≥60, diabetes, COPD, smoking, time to surgery≥4h, time to postoperative tube placement≥7d, albumin<30	–
Fan Yixin (2023) (14)	1	179	179	–	–	Logstic	Multifactor analysis	Age>73, Brinkman index≥200, history of lung disease, open surgery, PNI <45 points	Assigning values to variables
Zhang Yefan (2023) (17)	1	446	396	–	50	R software rms package	Multifactor analysis	Male, age ≥60 years, smoking, lung age, BMI	Nomograph
Dang Xinchen (2021) (18)	1	767	644	–	123	R software rms package	Multifactor analysis	Age, BMI, lung disease, diabetes, smoking, time of surgery, tumor site, surgical procedure	Nomograph
Xu Lei (2018) (19)	1	1016	646	160	210	Logstic	Multifactor analysis	Smoking index, surgical approach, POD1 neutrophil percentage, postoperative fasting glucose, anastomotic fistula, laryngeal reentrant nerve palsy	Formulas, Risk Scoring Scale
Chen Qiongyi (2023) (15)	8	408	302	75	31	Logistic、 Random Forest、 SVM、 XGBoost、 Light GBM、 KNN、 MLP、 AdaBoost	Multifactor analysis, LASSO, Boruta	Age, smoking, ASA score, serum cholinesterase, COPD, epidural analgesia, duration of surgery	Web Calculator
Bi Cen (2022) (20)	1	341	221	95	25	Logistic	Multifactor analysis	Age≥65, Brinkman index≥400, diabetes, open thoracic surgery, duration of surgery≥4, anesthesia by general anesthesia combined with epidural anesthesia, anastomotic fistulae	Risk Scoring Scale, formulas
Wang Wei (2022) (21)	1	78	78	–	–	R software rms package	R software glmnet, lasso	Differences in white blood cell counts before and after neoadjuvant chemotherapy, carbon monoxide diffusion capacity	Nomograph
Jin Donghui (2022) (22)	1	609	426	183	–	R software rms package	Multifactor analysis	Age, gender, surgical procedure, duration of chest tube placement, anastomotic leak, and laryngeal reentrant nerve palsy	Nomograph
Van (2014) (12)	2	206	185	–	21	cox	Multifactor analysis	Surgical access, age, male, history of COPD disease, number of lymph nodes removed	Scoring system
Ohkura (2020) (13)	8	10862	8715	2147	–	Logistic	Multifactor analysis	Age, esophageal cancer with gastrointestinal mesenchymal stromal tumor, male, diabetes mellitus, respiratory distress, preoperative ADL, COPD, preoperative pneumonia, peripheral vascular disease, stroke, weight loss>10%, Brinkman>600, creatinine >1.2, albumin <3.8 g/L, ALP>600U/L, blood urea nitrogen >20 mg/dL, PT <10 s	–
Li Shuang (2021) (25)	1	637	446	191	–	Logistic	lasso	Length of hospitalization, albumin, intraoperative bleeding, perioperative transfusion	Nomograph

PNI, Prognostic Nutritional Index; PCT, serum calcitoninogen; IL-1β, interleukin-1β; sTREM-1, soluble myeloid triggering receptor-1;CRP, C-reactive protein; COPD, chronic obstructive pulmonary disease; Brinkman index, smoking index; BMI, body mass index; -, not reported.

### 3.4 Model predictive performance

Regarding model performance, the reported discrimination in the model development research ranged from 0.627 to 0.850. Four studies evaluated the models using internal validation (13, 15, 20, 22), and four carried out external validation (16, 19, 23, 25). In the validation model studies, the area under the curve (AUC) values of the model validation groups exceeded 0.7, except for Jin’s model (22), which had an AUC value below 0.7. Although the discriminability of the model was not reported in van’s study (12), internal and external validation of the model was conducted by Weijs (31) and Seesing (32), and the mean value of the AUC was 0.94, which suggests that the model validation performance was stable. Wang (21) and Jin (22) reported only the calibration method of the model, while the study by Niu (24) and Fan (14) focused only on the sensitivity and specificity of the model. Notably, six studies (15–18, 21, 22) used calibration curves to assess the consistency between actual and predicted data, and three studies (15, 19, 20) chose the Hosmer-Lemeshow (H-L) goodness-of-fit test to assess model calibration. The risk prediction model performance is shown in **Table 3**.

**Table 3:** Performance of POP risk prediction model for EC (n=14).

Author (year)	AUC/ C-index	AUC/C-index		Sensitivity		Specificity		Calibration	Verification method
		Build	Verify	Build	Verify	Build	Verify		
Shi Peijun (2023) (16)	AUC	–	Model 1:0.856 Model 2:0.942	–	Model 1:77.78% Model 2:90.48%	–	Model 1:83.89% Model 2:90.05%	calibration curve	External
Li Cuihua (2023) (23)	AUC	–	0.876	–	–	–	–	–	External
Niu Lingjuan (2023) (24)	AUC	0.831	–	84.0%	–	67.5%	–	–	–
Fan Yixin (2023) (14)	AUC	0.770	–	60.7%	–	83.9%	–	–	–
Zhang Yefan (2023) (17)	C-index	0.713	–	–	–	–	–	calibration curve	–
Dang Xinchen (2021) (18)	C-index	0.782	–	–		–		calibration curve	–
Xu Lei (2018) (19)	AUC	0.721	0.736	73.9%		62.0%		H-L	External
Chen Qiongyi (2023) (15)	AUC	KGB:0.760 LR:0.722 LightGBM: 0.688 RF:0.809 AdaBoos: 0.717 SVM:0.627 MLP:0.646 KNN:0.726	–	KGB:71.6% LR:62.7% LightGBM: 64.1% RF:71.2% AdaBoost: 69.0% SVM:73.0% MLP:63.2% KNN:66.5%		KGB:74.1% LR:78.7% LightGBM: 70.3% RF:80.7% AdaBoost: 72.5% SVM:59.1% MLP:70.3% KNN:71.6%		calibration curve, H-L	Internal
Bi Cen (2022) (20)	AUC	0.781	0.771	70.4%	60.9%	73.7%	79.2%	H-L	Internal
Wang Wei (2022) (21)	C-index	0.850	–	–	–	–	–	calibration curve	–
Jin Donghui (2022) (22)	AUC	0.769	0.686	–	–	–	–	calibration curve	Internal
Van (2014) (12)	–	–	–	–	–	–	–	–	–
Ohkura (2020) (13)	C-index	0.632	–	–		–	–	–	Internal
Li Shuang (2021) (25)	C-index	0.802	0.763	–	–	–		–	External

-, not reported.

### 3.5 Risk of bias and applicability evaluation

All included studies had good applicability regarding the study population, predictors, and outcome domains. However, the overall risk of bias was high, indicating methodological issues in either the development or validation of the model. For risk of bias assessment, In the participant domain, 11 studies (13–15, 17–23, 25) were identified as having a high risk of bias, primarily due to their reliance on retrospective data. In the domain of predictor variables, all included studies were rated at low risk of bias. In the outcome domain, five studies (12, 17, 23–25) had uncertain forecast times. In addition, Ohkura Y (13) did not report diagnostic criteria for POP. In the area of analyses, two studies (13, 22) evaluated the results as having an unclear risk of bias, and the rest of the studies were assessed as having a high risk of bias in this area. The model risk of bias and applicability evaluations are shown in **Table 4**.

**Table 4:** Evaluations of the bias risk and applicability of the included models (n=14).

Author (year)	Risk of bias				applicability			Overall evaluation	
	Participant	Predictor	Outcome	Analysis	Participant	Predictor	Outcome	Risk of bias	applicability
Shi Peijun(2023) (16)	L	L	L	H	L	L	L	H	L
Li Cuihua(2023) (23)	H	L	U	U	L	L	L	H	L
Niu Lingjuan(2023) (24)	L	L	U	H	L	L	L	H	L
Fan Yixin(2023) (14)	H	L	L	H	L	L	L	H	L
Zhang Yefan(2023) (17)	H	L	U	H	L	L	L	H	L
Dang Xinchen(2021) (18)	H	L	L	H	L	L	L	H	L
Xu Lei(2018) (19)	H	L	L	H	L	L	L	H	L
Chen Qiongyi(2023) (15)	H	L	L	H	L	L	L	H	L
Bi Cen(2022) (20)	H	L	L	H	L	L	L	H	L
Wang Wei(2022) (21)	H	L	L	H	L	L	L	H	L
Jin Donghui(2022) (22)	H	L	L	U	L	L	L	H	L
Van(2014) (12)	L	L	U	H	L	L	L	H	L
Ohkura(2020) (13)	H	L	U	U	L	L	L	H	L
Li Shuang(2021) (25)	H	L	U	H	L	L	L	H	L

H, high risk of bias, high applicability risk; L, low risk of bias, low applicability risk; U, unclear.

## 4 Discussion

### 4.1 Efficacy of a prediction model for the POP in patients with EC and risk of bias analysis

This systematic review ultimately included 14 relevant studies reporting 23 models. The included models performed well in terms of applicability assessment and model differentiation. The mean AUC of model differentiation was 0.741.

However, the included studies also showed significant limitations in the evaluation of risk of bias, primarily attributable to the following reasons:(1) Inappropriate data sources: 78.57% of the studies in this review used a retrospective study design, and relevant factors, such as patients’ social support status, education level, and knowledge of the disease, could not be included, which resulted in an incomplete prediction factor prone to bias. (2) Insufficient sample size: In model development studies, 13 studies (12–22, 24, 25) had an event per variable (EPV) <20, and a low EPV can lead to overfitting of the model (11). In model external validation studies, the assessment of model performance may be overestimated when the outcome event (number of POP cases occurring) is <100 (33). However, in the external model validation, we found that only the study by Xu (19) fulfilled the sample size requirement. (3) Inappropriate treatment of continuous variables: three studies (14, 19, 20) converted predictors of continuous variables into categorical variables in model construction and did not state the basis for categorization. (4) Inappropriate treatment of missing data: Six studies (12, 15, 17–20) used the direct exclusion method to deal with missing data, which may lead to the researchers ignoring the potential predictor variables in the excluded subjects, thus affecting the final data quality. (5) Incomplete assessment of model performance: Six studies (12–14, 23–25) did not report the calibration of the model, which may not be able to assess the actual prediction accuracy of the model and cause bias. (6) Only four studies (13, 15, 20, 22) performed internal validation and four studies (16, 19, 23, 25) performed external validation; the lack of internal validation makes the problem of overfitting easy to be ignored (34); furthermore, it is detrimental to the clinical promotion of the model’s application.

At present, there is still a problem of heavy development but light validation in the field of predictive model construction, and some studies only perform model construction and ignore the importance of the actual clinical application of predictive models. To improve reporting and study quality, the PROBAST (11) assessment criteria can be useful. In terms of study design, prospective or randomized controlled studies should be conducted according to these assessment criteria, and a sufficiently large study sample size should be selected to reduce the difference between the study population and the actual population. In terms of data processing, variables and missing data should be treated rigorously. Commonly used methods for processing missing data include interpolation, deletion, and weighting (35), which are reasonable methods for accurately reflecting the characteristics of the population at risk. Furthermore, the use of objective tools is necessary to assess model performance, validate the model internally and externally to ensure the model’s practicability and popularization in clinical application, enhance the persuasiveness and accuracy of the study, and reduce the risk of bias.

### 4.2 Predictor analysis of a predictive model for POP risk in patients with EC

Because of differences in the diagnosis of POP, the predictive variables included, and research organizations, the risk factors that induce POP are both different and common across studies.In this study, the five most frequent predictor variables of risk for POP in patients with esophageal cancer were found to be: smoking, age, respiratory disease (COPD), diabetes mellitus, and surgical procedure (Open chest surgery).

Elderly individuals are one of the populations most affected by POP, and with age, there are degenerative changes in body functions. One study (36) found that the risk of POP was 2.68 times higher in the older population than in those aged <60 years. In older adults, lung tissue defenses are lower. Furthermore, immunity declines with age. The immune response reduces alveolar macrophage function, which can lead to the development of lung infections (18). Smoking contributes to the development of respiratory symptoms, and the risk of respiratory symptoms is proportional to the duration and amount of smoking (37). Harmful substances in tobacco irritate the respiratory tract, damage the respiratory mucosa, and affect the cilia’s ability to clean, resulting in excessive mucus secretion that cannot be cleared by lung tissues, thus increasing the risk of lung infections (38). Guidelines (39) recommend that patients undergoing esophagectomy should quit smoking for at least 4 weeks before surgery to reduce the incidence of lung disease. Furthermore, healthcare professionals should encourage perioperative patients to quit smoking and perform respiratory exercises to prevent POP. Moreover, patients with chronic obstructive pulmonary disease, emphysema, and other chronic respiratory diseases before surgery have high amounts of secretions in the respiratory tract that are not easy to cough up, which increases the risk of POP to a certain extent (15). Patients with diabetes mellitus should actively control their blood glucose levels before surgery, and a hyperglycemic environment is conducive to bacterial reproduction and induces infection (16). Relevant guidelines (39) recommend minimally invasive luminal surgery as the primary treatment modality for patients undergoing EC surgery. Compared with minimally invasive luminal surgery, traditional surgery is traumatic, and postoperative patients often do not dare to cough due to wound pain; therefore, they cannot expel sputum in time, thus inducing POP (20). Clinical staff should pay attention to the perioperative assessment of elderly patients, preoperative patients with underlying lung diseases and diabetes mellitus, and smoking patients and efficiently identify high-risk groups. For high-risk patients, healthcare professionals should provide detailed perioperative education, supervise patients to quit smoking, and control blood glucose levels to improve the patient’s awareness of the risk of lung infection and select the optimal surgical treatment plan according to the patient’s condition.

Note that most risk prediction studies have neglected the potential impact of perioperative laboratory indicators. The laboratory indicator that appeared most frequently in the studies included in this article was albumin, and only three studies (13, 24, 25) have reported that a decrease in the body’s albumin levels not only caused a decrease in the patient’s immunity but also was accompanied by a decrease in plasma osmolality, which induces lung infection (25). Note that Xu (19) identified the significance of postoperative fasting blood glucose levels ≥7.1 in predicting POP development. Most studies have primarily focused on history of diabetes mellitus as a crucial risk factor for POP, often overlooking the harmful effects of transient hyperglycemia. Transient hyperglycemia is not diabetes mellitus; it may indicate stress-induced hyperglycemia, which compromises leukocyte bactericidal capacity and elevates the risk of POP (40). Therefore, perioperative laboratory indicators are also important predictors of the occurrence of POP in patients with EC; however, they have been ignored by researchers because of limited data acquisition caused by differences in the types of studies and medical equipment. Relevant risk factors should be comprehensively and systematically included in the future, aiming to prevent the occurrence of POP to the greatest extent possible.

### 4.3 Future trends and challenges in the predictive modeling of POP risk in patients with EC

Research on POP risk prediction models for patients with EC started late, and there has been a rapid development trend in the past 2 years; however, the effectiveness of these models varies. External validation studies on most developed models are limited. Among the models included in this study, only 28.57% of the studies (16, 19, 23, 25) conducted external validation. Weijs (31) and Seesing (32) performed internal and external validation of van’s scoring system with outstanding predictive results (12), offering novel insights for the clinical diagnosis of POP. However, note that this validation was limited to the United States and the Netherlands, necessitating further investigation into the applicability of the model in other regions. Ohkura conducted a multicenter study using data from the Japanese National Clinical Data Bank (13); however, because of a lack of standardized surgical teams, the model exhibited poor predictive efficacy. The nomogram prediction model of Shi had good model efficacy in external validation (16), which contained nine predictive entries; however, serological indicators, such as PCT, IL-1β, sTREM-1, and CD4^+^/CD8^+^, among the predictors under study, were difficult to obtain and expensive, which was not conducive to the generalization of the predictive factors in clinical practice. The model of Li (25) contained four predictive entries: hospitalization time, albumin, intraoperative bleeding, and perioperative transfusion, which are relatively easy to obtain in clinical work and are more likely to be adopted by healthcare professionals. However, Li’s model was only externally validated, and external validation is required to strengthen the stability and persuasiveness of the model based on internal validation. Chen (15) and Fan (14) analyzed the risk factors of vulnerable populations by constructing a POP risk prediction model for elderly patients with EC, suggesting that future researchers should consider conducting risk prediction modeling studies for different target populations. With the rise of big data applications, researchers have begun to attempt developing modeling techniques. Chen (15) constructed eight models based on machine learning methods; however, the model lacked external validation to assess its accuracy, which encourages future researchers to try new modeling techniques based on machine learning to build models with stronger predictive capabilities.

Six diagnostic criteria for POP exist for the studies included in this study. Fortunately, We found commonalities in these diagnostic criteria based on authoritative, evidence-based evidence. These diagnostic criteria are based on the clinical signs of infection, such as increased leukocyte count, increased sputum in the respiratory tract, increased body temperature, and imaging tests. We remain hopeful that in the future, there will be a globally recognized and uniform diagnostic standard for POP that will facilitate the predictive power and generalizability of inter-model comparisons.

### 4.4 Recommendations for future research

Currently, the predictive variables of POP risk among patients undergoing EC surgery are not well unified. In the future, studies can be conducted based on the risk factors for POP occurrence in different surgical procedures or special populations; more high-quality models can be constructed; and multicenter and large-sample internal and external validation can be conducted to improve the persuasiveness and accuracy of the models. Furthermore, researchers must consider the feasibility and simplicity of models in clinical practice, identify potential problems in model application early, and improve them. The perioperative training of healthcare workers is also an issue that should not be ignored. Understanding the acceptability and workload of healthcare workers and encouraging them to propose limitations of model application and improve them will enhance the trust of clinical workers on the theoretical basis and facilitate the practical application of the model.

## 5 Conclusion

In conclusion, the 14 studies of POP risk prediction models for patients with EC included in this study had high model efficacy and applicability; however, the overall risk of bias was high, different predictor variables among different studies were not quantitatively analyzed, and most models were not externally validated, resulting in poor model generalization. Future studies may adopt prospective, randomized controlled methods for model construction and internal and external validation. Multicenter and large-sample validation studies based on existing models should be conducted to enhance the generalizability of the models in clinical applications. The use of machine learning and other emerging technologies must be considered to develop predictive models with good predictive performance, high accuracy, and ease of operation, to provide a theoretical basis for their clinical applications.

## Data availability statement

The raw data supporting the conclusions of this article will be made available by the authors, without undue reservation.

## Author contributions

YJ: Writing – original draft, Writing – review & editing. ZL: Data curation, Writing – review & editing. WJ: Data curation, Writing – review & editing. TW: Formal analysis, Writing – review & editing. BC: Supervision, Writing – review & editing.
